# Deoxyelephantopin from *Elephantopus scaber* Inhibits HCT116 Human Colorectal Carcinoma Cell Growth through Apoptosis and Cell Cycle Arrest

**DOI:** 10.3390/molecules21030385

**Published:** 2016-03-21

**Authors:** Chim Kei Chan, Gomathi Chan, Khalijah Awang, Habsah Abdul Kadir

**Affiliations:** 1 Biomolecular Research Group, Biochemistry Program, Institute of Biological Sciences, Faculty of Science, University of Malaya, 50603 Kuala Lumpur, Malaysia; chimkei@gmail.com; 2 Department of Chemistry, Faculty of Science, University of Malaya, 50603 Kuala Lumpur, Malaysia; cg_gramaf@hotmail.com (G.C.); khalijah@um.edu.my (K.A.)

**Keywords:** deoxyelephantopin, apoptosis, cell cycle, colorectal cancer, *Elephantopus scaber*

## Abstract

Deoxyelephantopin (DET), one of the major sesquiterpene lactones derived from *Elephantopus scaber* was reported to possess numerous pharmacological functions. This study aimed to assess the apoptosis inducing effects and cell cycle arrest by DET followed by elucidation of the mechanisms underlying cell death in HCT116 cells. The anticancer activity of DET was evaluated by a MTT assay. Morphological and biochemical changes were detected by Hoescht 33342/PI and Annexin V/PI staining. The results revealed that DET and isodeoxyelephantopin (isoDET) could be isolated from the ethyl acetate fraction of *E. scaber* leaves via a bioassay-guided approach. DET induced significant dose- and time-dependent growth inhibition of HCT116 cells. Characteristics of apoptosis including nuclear morphological changes and externalization of phosphatidylserine were observed. DET also significantly resulted in the activation of caspase-3 and PARP cleavage. Additionally, DET induced cell cycle arrest at the S phase along with dose-dependent upregulation of p21 and phosphorylated p53 protein expression. DET dose-dependently downregulated cyclin D1, A2, B1, E2, CDK4 and CDK2 protein expression. In conclusion, our data showed that DET induced apoptosis and cell cycle arrest in HCT116 colorectal carcinoma, suggesting that DET has potential as an anticancer agent for colorectal carcinoma.

## 1. Introduction

Colorectal cancer is the third ranked deadliest malignancy in the world. Chemotherapy is the current mode of treatment for colorectal cancer but it has limited efficacy with escalating side effects. Plants are an important source in the development of naturally-derived pharmaceutical agents for the treatment or prevention of various pathologies. To date, plants continue to contribute novel leads for drug discovery targeting a multitude of ailments, including cancer [1]. Currently, more than 50% of all approved drugs are natural products and their derivatives [2].

Phytochemicals have been reported to mediate diverse mechanisms against cancer including apoptosis, autophagy and cell cycle modulation. Apoptosis induction is a classical approach in initiating cell death characterized by biochemical (phosphatidylserine externalization, depolarization of mitochondrial membrane potential and caspase activation) and morphological hallmarks (DNA fragmentation, cell shrinkage and chromatin condensation) [3,4].

Various cytotoxic agents and DNA damaging inducers are capable of arresting cell cycle at the G0/G1, S or G2/M phase, consequently resulting in apoptosis induction in cancer [5,6,7]. Cell cycle is orchestrated by concerted actions of cyclin/cyclin-dependent kinase (CDK) in association with cyclin proteins. Cyclin D-CDK4/CDK6 and cyclin E-CDK2 navigate G1 transition via the restriction point, which lead to the cell cycle progression [8]. The cyclin E-CDK2 and cyclin A-CDK2 are essential complexes involved in the initiation and progression of the S phase [9] while the cyclin B-CDK1 complex regulates progression of G2 and mitosis [10]. These complexes in turn, are regulated by the cyclin-dependent kinase inhibitors (CDKIs), including p21^Waf1/Cip1^, a crucial regulator of cell cycle progression and apoptosis. The functional role of p21 can be initiated either by p53-dependent or -independent mechanisms [11,12].

*Elephantopus scaber* Linn., commonly known as Elephant’s foot, (Asteraceae) is a scabrous herb distributed on various continents, including Asia, Europe, South America and Africa. According to traditional claims, the leaves and roots are used for fever, diarrhea, dysentery, cancer and dysuria [13,14]. *E. scaber* is a rich source of sesquiterpene lactones, including deoxyelephantopin (DET) and isodeoxyelephantopin (isoDET), that have been used in Ayurveda medicine in India for the treatment of cancer and leukemia [15]. Pharmacological studies have demonstrated the antitumor effects of on breast, nasopharyngeal, lung, cervical, Dalton lymphoma ascitis and prostate cancer [16,17,18,19,20]. DET induced apoptosis via intrinsic and extrinsic pathway as well as cell cycle arrest in human nasopharyngeal carcinoma epithelial CNE cells. Additionally, isoDET was shown to induce apoptosis in T47D breast carcinoma, A549 lung carcinoma and KBM-5 chronic myeloid leukemia [21,22].

Previously, we have found the ethyl acetate fraction from *E. scaber* could inhibit the growth of the human colorectal cancer cell line HCT116 [23]. In continuation of that work, here we aimed to isolate and identify the active compounds from ethyl acetate fraction of *E. scaber* based on its ability to inhibit HCT116 cell growth *in vitro*. We identified two anticancer compounds, deoxyelephantopin (DET) and isodeoxyelephantopin (isoDET). The profound cytotoxicity of DET against HCT116 cells indicates that this compound maybe valuable for colorectal cancer therapy. However, the HCT116 cell growth inhibition activities of DET, as well as its apoptosis inducing action and underlying molecular mechanisms have never been reported before. The apoptosis inducing effect of DET on HCT116 cells has been reported once previously and was only based on morphological studies [20]. Towards this end, the current study investigated the effect of DET in HCT116 cells on the cell cycle and apoptosis regulation. These results may help the future development of new colorectal cancer therapies.

## 2. Results

### 2.1. Isolation and Identification of the Bioactive Compounds, Deoxyelephantopin and Isodeoxyelephantopin via a Bioassay-Guided Approach

**Figure 1:**
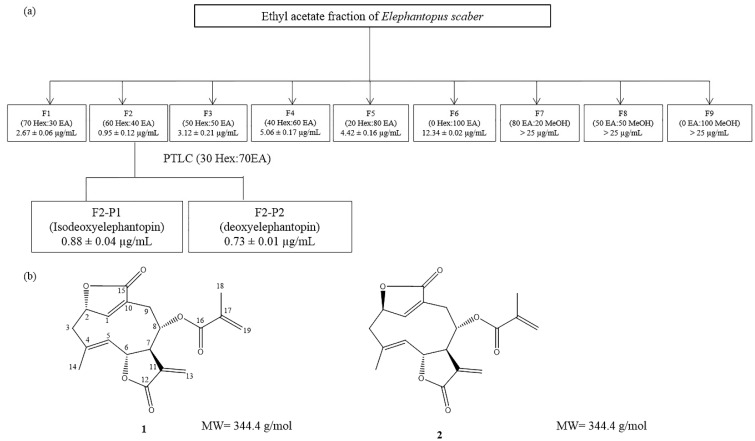
Bioassay guided isolation and identification of bioactive phytochemicals from ethyl acetate fraction of *E. scaber*. (**a**) Flow chart showed the bioassay guided isolation of bioactive phytochemicals. Each fractions was dissolved in DMSO and the final concentration of DMSO was below 0.5% *v*/*v* in all experiments. All fractions were assessed for its cytotoxic effect on HCT116 cells using MTT assay. The IC_50_ values were expressed as mean ± S.E. (*n* = 9); (**b**) Chemical structures of isodeoxyelephantopin (**1**) and deoxyelephantopin (**2**).

Our previous study demonstrated that the ethyl acetate fraction of *E. scaber* exerted the most potent cytotoxic effect on HCT116 cells [23]. Hence, we proceeded with the isolation of its bioactive compounds via a bioassay guided approach (Figure 1). Compounds **1** and **2** were isolated from the active fraction F2. The structures of compounds **1** and **2** were elucidated using spectroscopic analysis (^1^H-, ^13^C-NMR and HRESI-MS), and by comparison of the obtained spectral data with literature values. Compound **1** was identified as isodeoxyelephantopin (isoDET), by comparison of its ^1^H- and ^13^C-NMR data with the literature values of isodeoxyelephantopin (isoDET) [24] (Table 1). The structure of compound **1** was further confirmed as isoDET by HRESI-MS, in a positive mode, which revealed a molecular ion peak at *m*/*z* 345.1339 [M + H]^+^, which corresponds to a molecular formula of C_19_H_20_O_6_. Compound **2** was identified as deoxyelephantopin (DET), by comparison of its ^1^H- and ^13^C-NMR data with literature values of DET [25] (Table 2). The structure of compound **2** was further confirmed as DET by HRESI-MS, in positive mode, which revealed the molecular ion peak at *m*/*z* 345.1332 [M + H]^+^, which corresponds to a molecular formula of C_19_H_20_O_6._

### 2.2. DET and isoDET Inhibited the Proliferation of HCT116 Cells

**Figure 2:**
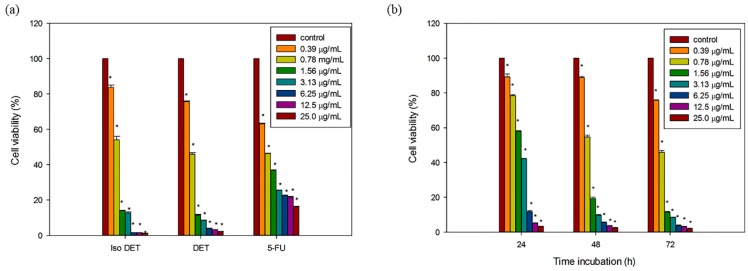
Effect of deoxyelephantopin and isodeoxyelephantopin on the cell viability of HCT116 cells. (**a**) The bar chart showed the percentage of cell viability after treatment with different concentrations of DET, isoDET and 5-fluorouracil ranging from 0.39–25.0 µg/mL on HCT116 cells for 72 h; (**b**) The bar chart showed the cytotoxicity of DET at different time incubation (24, 48 and 72 h) on HCT116 cells. The data expressed as mean ± S.E. of three independent experiments (*n* = 9). Asterisks indicate significantly different value from control (* *p* < 0.05).

DET and isoDET were assessed for their cytotoxicity on the HCT116 human colorectal carcinoma cells and CCD841CoN normal colon cells using MTT assays. Treatment of HCT116 cells with DET and isoDET caused a dose-dependent reduction of cell viability at 72 h (Figure 2a).

The IC_50_ values of DET and isoDET for HCT116 cells were 0.73 ± 0.01 µg/mL (2.12 µM) and 0.88 ± 0.02 µg/mL (2.56 µM), respectively (Table 3). DET was relatively less cytotoxic to the CCD841CoN normal cells (21.69 ± 0.92 µg/mL (60.02 µM)) when compared to HCT116 cells, revealing a 30 fold difference in cytotoxicity. Interestingly, 5-fluorouracil exhibited a comparable IC_50_ value of 0.73 ± 0.02 µg/mL (5.0 µM) in HCT116 cells. Treatment of HCT116 cells with DET caused significant time-dependent reduction in cell viability with the IC_50_ values of 2.36 ± 0.02, 0.9 ± 0.02 and 0.73 ± 0.01 µg/mL at 24, 48 and 72 h, respectively (Figure 2b).

### 2.3. Apoptotic Morphological Changes by DET

**Figure 3:**
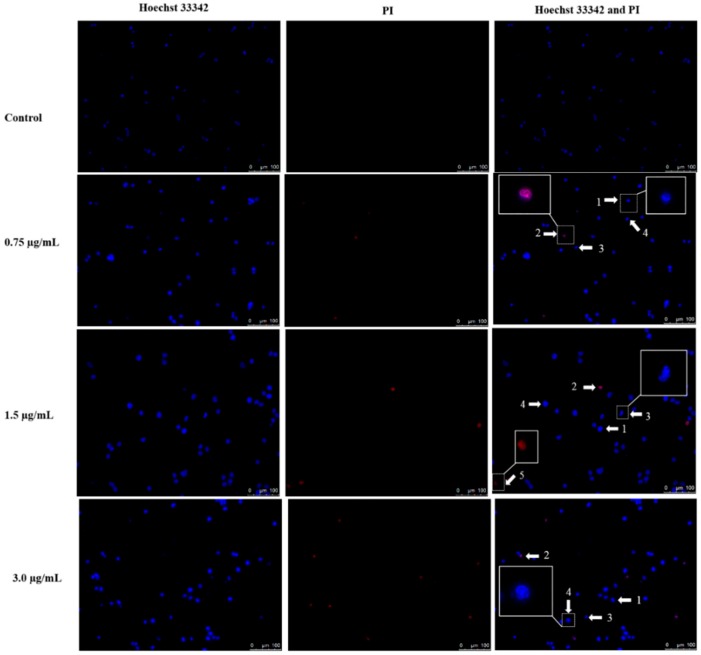
Effect of deoxyelephantopin on nuclear morphological changes of HCT116 cells. After exposure to 0.75 µg/mL, 1.5 µg/mL and 3.0 µg/mL of DET at 24 h, cells were stained with Hoechst 33342 and PI followed by observation under fluorescence microscope. Magnification: 200× Arrow 1 chromatin condensation, 2 late apoptosis, 3 cell shrinkage, 4 DNA fragmentation, 5 necrosis.

Apoptotic morphological changes can be detected by using Hoechst 33342/PI staining after treatment with DET on HCT116 cells. Fluorescence microscopic data on morphological changes of HCT116 cells are shown in Figure 3. As illustrated in Figure 3, fluorescence microscopic data indicated an apoptosis-inducing effect of DET in a dose-dependent manner. In the untreated control, HCT116 cells were normally round in shape and homogeneously stained, with intact chromatin in the nuclei. However, following treatment with increasing concentrations of DET for 24 h, an increased number of HCT116 cells with chromatin condensation, cell shrinkage, DNA fragmentation, apoptotic bodies and necrosis along with a decreased number of HCT116 cells with normal morphology were both observed (Figure 3).

### 2.4. Externalization of Phosphatidylserine by DET

**Figure 4:**
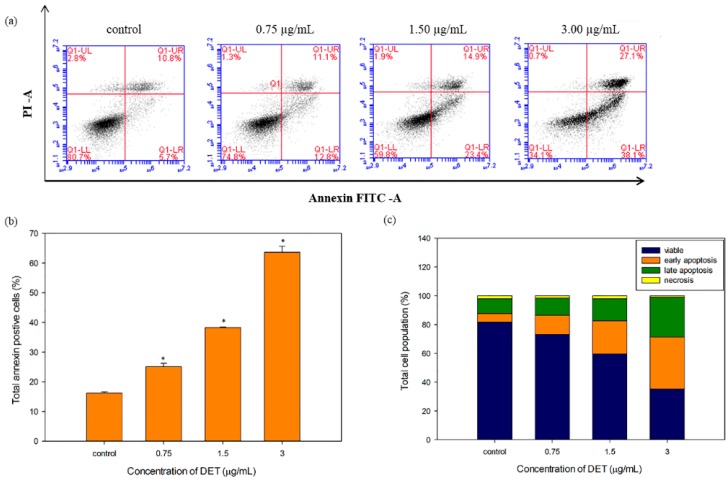
Externalization of phosphatidylserine induced by different concentrations of deoxyelephantopin (0.75–3.0 µg/mL) detected using Annexin V-FITC/PI staining at 24 h. (**a**) displayed the flow cytometric fluorescence patterns; (**b**) The bar chart showed the percentage of Annexin V positive cells; (**c**) The bar chart summarized the percentage of viable, early apoptotic, late apoptotic and necrotic cells. The data expressed as mean ± S.E. from three individual experiments. Asterisks indicate significantly different value from control (* *p* < 0.001).

Phosphatidylserine externalization, a biochemical hallmark of the early apoptosis, can be confirmed by annexin V-FITC/PI staining and flow cytometry. DET treatment of HCT116 cells distributed the cells into different phases including viable (annexin^−^/PI^−^), early apoptosis (annexin^+^/PI^−^), late apoptosis (annexin^+^/PI^+^), and necrosis (annexin^−^/PI^+^). As shown in Figure 4, the proportion of apoptotic cells (early and late apoptotic phases) significantly increased and peaked at 63.63% ± 2.03%, corresponding to the highest concentration (3.0 µg/mL) of DET. Even the lowest concentration (0.75 μg/mL) of DET induced significant accumulation of apoptotic cells compared to the untreated control. Further, the proportion of viable cells also decreased with increasing concentrations of DET.

### 2.5. Modulatory Effects of DET on Apoptosis-Related Protein Expression

**Figure 5:**
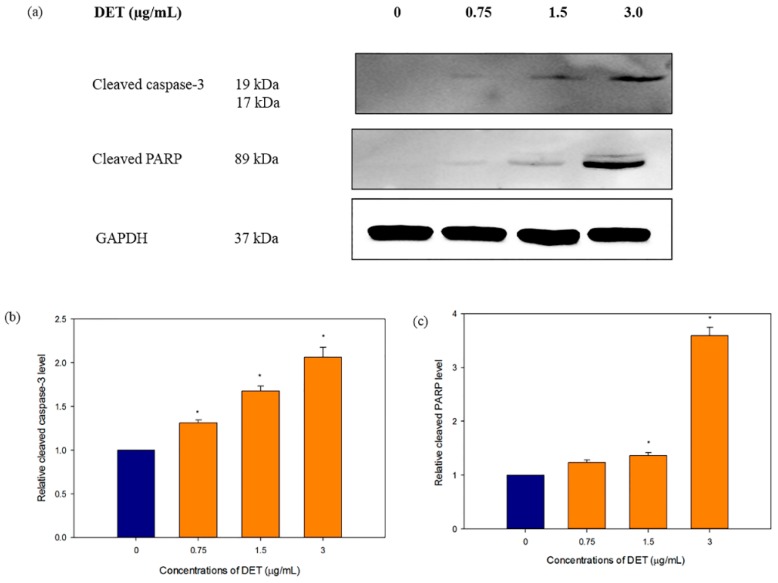
Effect of deoxyelephantopin on the apoptosis-related protein expressions in HCT116 cells. HCT 116 cells were treated with different concentrations of DET (0.75, 1.5 and 3.0 µg/mL) for 24 h. (**a**) Representative image of cleaved caspase-3 and cleaved PARP expression level with their respective western blot band intensity; (**b**) The bar chart showed the relative protein expression of cleaved caspase-3 level; (**c**) The bar chart showed the relative protein expression of cleaved PARP. The data expressed as mean ± S.E. from three individual experiments. Asterisks indicate significantly different value from control (* *p* < 0.001).

To decipher the apoptosis-inducing mechanism of DET in HCT116 cells, western blotting was used to evaluate the apoptosis-related protein expression of cleaved caspase-3 and cleaved PARP. During the apoptosis, activation of caspase-3 can result in proteolytic degradation of PARP which is one of the known apoptotic enzyme indicator. Consistent with the apoptosis data, treatment with DET caused caspase-3 and PARP cleavage (Figure 5a). As shown in Figure 5, DET caused a dose-dependent upregulation of cleaved caspase-3 and cleaved PARP protein expression when the HCT116 cells were treated with varying concentrations of DET (0.75, 1.5 and 3.0 µg/mL). Taken together, the results suggested that DET mediated cell death via apoptosis in HCT116 cells.

### 2.6. Induction of Cell Cycle Arrest by DET

**Figure 6:**
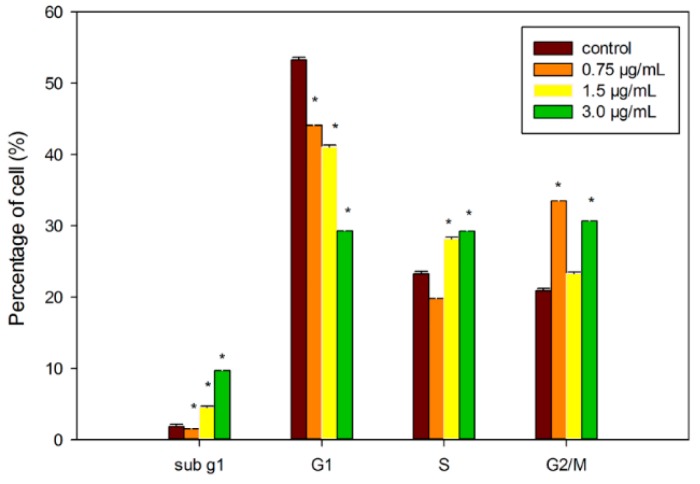
Effect of DET on the cell cycle distribution of HCT116 cells. An accumulation of S-phase cell distribution after treatment with varying concentrations of DET (0.75, 1.5 and 3.0 µg/mL) in HCT116 cells at 24 h. The data expressed as mean ± S.E. from three individual experiments. Asterisks indicate significantly different value from control (* *p* < 0.001).

The cell cycle progression in DET-treated cells was also examined by flow cytometry. The results revealed that DET induced cell cycle arrest at S phase in the HCT116 cells. There was a significant dose-dependent accumulation of cells in the S phase when exposed to 1.5 and 3.0 µg/mL, accounting for 28.17% ± 0.23% and 29.25% ± 0.21%, respectively at 24 h (Figure 6). Concurrently, DET caused a corresponding reduction of cells at G1 and G2/M phases and a significant increased population of hypodiploid (sub G1/apoptosis). Therefore, our results suggest that there was a blockage at the S phase, which led to cell growth inhibition. Additionally, the sub G1 peaks were further substantiated by the corresponding externalization of phosphatidylserine suggesting that apoptosis was mediated by inhibition of the cellular mitosis.

### 2.7. Modulatory Effects of DET on Cell Cycle-Related Protein Expression

**Figure 7:**
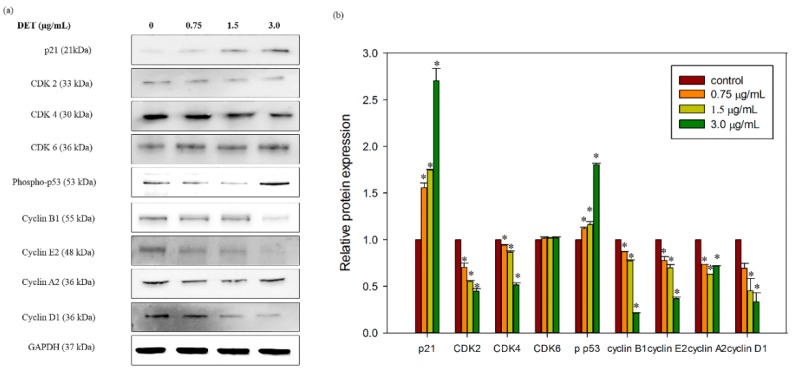
Effect of DET on cell cycle-related protein expressions in HCT116 cells. HCT116 cells were treated with different concentrations of DET (0.75, 1.5 and 3.0 µg/mL) at 24 h. (**a**) Representative image of p21, phospho-p53, CDK2, CDK4, CDK6, cyclin B1, cyclin E2, Cyclin A2 and Cyclin D1 expression level with their respective western blot band intensity; (**b**) The bar chart showed the relative protein expression levels. The data expressed as mean ± S.E. from three individual experiments. Asterisks indicate significantly different value from control (* *p* < 0.001).

Next, we also verified the effect of this sesquiterpene on the cell cycle-related protein expression in HCT116 cells as DET induced cell cycle arrest in S phase. The results demonstrated that DET induced a significant downregulation in the protein expression of CDK4, CDK2 and cyclin A2, B1, E2 and D1 which are essential molecules in regulating cell cycle progression (Figure 7a). This reduction of the protein expression indicated dose dependency of DET (Figure 7b). In contrast, CDK6 was found to remain constant after treatment with DET (Figure 7b). In addition, the expression level of cell cycle inhibitor p21 and phospho-p53 was upregulated in the presence of different concentrations of DET at 24 h in HCT116 cells (Figure 7b). These results further corroborated the flow cytometry findings indicating that DET arrested cells at the S phase.

## 3. Discussion

Sesquiterpene lactones, the active constituents of various herbal plants, have been reported to possess a manifold of health benefits including anti-inflammatory and anticancer effects. As such, the anticancer effect of sesquiterpene lactones has raised great attention among the researchers [26]. For instance, costunolide from *Magnolia sieboldii* induced apoptosis via ROS-mediated activation of JNK in *in vitro* and *in vivo* models of human leukemia cells [27]. Another sesquiterpene lactone, deoxyelephantopin (DET), showed apoptosis induction via multiple apoptotic signaling pathways in *in vitro* and *in vivo* models of human TS/A mammary adenocarcinoma cells [18]. In the present study, however, we have safely concluded the apoptosis inducing effect of DET and delineated its underlying mechanisms in HCT116 cells.

According to our previous findings, the ethyl acetate fraction of *E. scaber* exerted the greatest cytotoxic effect towards HCT116 human colorectal cancer cells [23]. Thus in this study, we used a bioassay-guided approach via MTT assay to isolate the bioactive compounds responsible for the observed cytotoxic effects. Our current findings indicated that DET from *E. scaber*, exhibited a significantly lower IC_50_ value than isoDET in HCT116 cells. Interestingly, DET also showed a considerably lower cytotoxicity in normal colon cells CCD841 CoN. Evidently, DET was a more potent growth inhibitor as compared to isoDET, thus, it was selected for further investigations on the underlying mechanisms.

Cell death is the outcome of three well known dynamic cellular activities such as apoptosis, autophagy and necrosis [28]. Apoptosis is a ubiquitous mechanism which involves nuclear shrinkage, chromatin condensation, DNA fragmentation, phosphatidylserine externalization, depolarization of mitochondria membrane potential and activation of caspase [3]. By fluorescence microscopy, a pronounced alteration in nuclear morphology was observed in a dose-dependent manner when DET-treated HCT116 cells were stained with Hoechst 33342/PI. Apoptotic features including DNA fragmentation, nuclear shrinkage and chromatin condensation markedly increased with increasing concentrations of DET in treated cells at 24 h. The dissipation of asymmetrical plasma membrane resulted in exposure of phosphatidylserine to the outer leaflet of plasma membrane indicating detection of early stage of apoptosis by Annexin V-FITC/PI assay [29]. In the early apoptosis, the cell membrane remains intact whereas in the late apoptosis and necrosis, the plasma membrane loses its asymmetrical integrity and increases the permeability to non-cell permeable dye such as propidium iodide [29]. The conclusive evidence that DET induced apoptosis was inferred from the morphological analysis and characterization of phosphatidylserine externalization in DET-treated HCT116 cells. DET did not significantly induce necrotic cell death.

Caspases play a pivotal role in mediating the process of apoptosis which is one of the major routes in combating cancer. Activated caspase-3 is known as an executioner caspase which can trigger apoptosis by cleaving substrates including PARP, lamin and other related substrates, ultimately orchestrates the biochemical and morphological characteristic changes in apoptotic cells. Activation of caspase cascade leads to fragmentation of PARP which prevents DNA repair cycles resulting in DNA fragmentation [30,31]. Thus, our findings indicated that DET induced activation of caspase-3 with a concomitant elevation of PARP cleavage. The cleavage of PARP was accompanied by increasing DNA fragmentation upon exposure to DET. Collectively, these data further suggested that DET executed cell death in HCT116 cells through apoptosis.

Dysregulation of cell cycle is a key feature of tumor cells and hence targeting the cell cycle is an important approach in cancer therapy [32]. In the present study, we observed that DET could induce S phase arrest in HCT116 cells. Arguably the most crucial phase of the cell cycle are the S phase, the occurrence of DNA replication and M phase, the cell division into two daughter cells. Reported anticancer agents known to arrest cell cycle at S phase in different types of cancer cells include resveratrol and cisplatin [6,33]. Similarly, treatment with DET in HCT116 cells was found to induce S phase arrest thus preventing the transition from S phase to G2/M phase. Accordingly, the ability of DET to block the S-G2 transition has been reported previously in human CNE nasopharyngeal cells [34]. Cell cycle machinery is controlled by cyclin-dependent kinase (CDK), cyclins and CDK inhibitory proteins. CDK inhibitory protein, p21, an essential signaling molecule in regulating the cell cycle progression by interacting directly with CDK/cyclin complexes. The expression of CDK inhibitory protein p21 has been exploited in the development of chemotherapeutic drugs that disrupt tumorigenesis by halting the cell cycle in cancer cells. The present results indicated a dose-dependent upregulation of p21 in DET-treated cells. Besides p21, the tumor suppressor p53 also could induce cell cycle arrest and apoptosis which contributes to conserve genome stability integrity in response to wide range of cellular stress and DNA damage [35]. G1/S checkpoint is putatively initiated with the activation of p53, followed by a canonical upregulation of p21 in response to DNA damage [36,37]. The increased levels of phospho-p53 and p21 inhibited both the cyclin E and cyclin A/cdk2 expression which are required for transitioning G1 into S phase. For instance, oridonin induced cell cycle arrest in the S phase via activation of p53 and p21 leading to apoptosis [38]. Our results demonstrated that DET also upregulated p53 expression. Thus, our findings suggested that p53 and p21 might execute their functions by suppressing the kinase of CDK-cyclin complexes to induce S phase arrest caused by DET. It has been reported that the cell cycle regulatory protein expression of cyclin A, cyclin E, cyclin D, Cdk4, Cdk6 and Cdk2 were inhibited by the activation of p21 in human cancer cells [39]. For instance, a study demonstrated that hinokitiol, a tropolone-related natural compound, downregulated the expression of cyclin A, cyclin E and Cdk2 via activation of p21 in HCT116 human colon cancer cells [40]. The expression of cyclin B1, cyclin D1 and cyclin A was decreased by resveratrol in SW480 human colorectal adenocarcinoma cells [6]. Consistent with this notion, our findings demonstrated that DET induced S phase arrest through downregulation of CDK2, CDK4 cyclin A2, cyclin D1, cyclin E2 and cyclin B1 protein expression. Interestingly, DET also showed an induction of cell cycle arrest at sub G1 phase in HCT116 cells which indicated the accumulation of apoptotic cells population. The accumulation of the apoptotic cells was supported by the increase of the apoptotic markers including cleavage of caspase-3 and PARP. Apoptosis induction was correlated with caspase-3 activation and PARP cleavage in HCT116 cells.

## 4. Materials and Methods

### 4.1. Plant Material

The leaves of *E. scaber* were obtained, extracted and fractionated as previously described. A voucher specimen (No. KLU47976) was deposited at the herbarium of Institute of Biological Sciences, Faculty of Science, University of Malaya, Kuala Lumpur, Malaysia [23].

### 4.2. Isolation of Active Phytochemicals from the Ethyl Acetate Fraction of E. scaber via a Bioassay-Guided Approach

Based on the previous results [23], a portion of the active ethyl acetate fraction (11 g) was subjected to column chromatography (CC) fractionation over silica gel (60–200 mesh) using a dry packing method. The column was eluted with a gradient solvent system of hexane–ethyl acetate (7:3 to 0:10) and ethyl acetate–methanol (8:2 to 0:10) to yield nine fractions (F1–F9, Figure 1). All fractions were evaporated by using a rotary evaporator. The fractions were then dissolved in DMSO with the final concentration of 0.5% *v*/*v* DMSO. MTT assay was performed on each fraction. Fraction F2 showed the most potent cytotoxicity (0.95 ± 0.12 µg/mL), consequently fraction F2 was further purified by preparative thin layer chromatography (PTLC) and eluted with hexane–ethyl acetate (3:7), three times, which yielded compound **1** (0.18 g, R_f_ = 0.75) and compound **2** (0.29 g, R_f_ = 0.70). Both of these compounds were subjected to the MTT assay.

### 4.3. Cell Culture

HCT116 human colorectal cancer cells and CCD841-CoN normal cells were obtained from the American Type Culture Collection (ATCC, Manassas, VA, USA). HCT116 cells and CCD841-CoN normal cells were cultured in RPMI-1640 and EMEM media respectively, with 10% *v*/*v* heat-inactivated fetal bovine serum (FBS), 100 µg/mL penicillin streptomycin and 50 µg/mL amphotericin B at 37 °C in 5% CO_2_ incubator.

### 4.4. Cytotoxic Effect Determined by MTT Assay

To investigate the cytotoxic effect of each fraction and compounds, the MTT assay was utilized. Cells were seeded and exposed to different concentrations (0.38–25 µg/mL) of each fraction which was dissolved in DMSO. 5-fluorouracil (5-FU) was used as positive control. Untreated cells (negative control) were treated with vehicle dimethyl sulfoxide (DMSO). The final concentration of DMSO was below 0.5% *v*/*v* in all experiments. MTT (5 mg/mL) was then added to each well after treatment and incubated for another 4 h at 37 °C. Subsequently, the medium was discarded and replaced by 150 µL of DMSO to dissolve the formazan crystals followed by quantified at 570 nm and 650 nm as a background using a microplate reader (Asys UVM340, Biochrom, Eugendorf, Austria). The percentage of cell viability was calculated according to the following equation: Cell viability percentage (%) = (Absorbance of treated cells/absorbance of untreated cells) × 100%.

### 4.5. Induction of Nuclear Morphological Changes by Hoechst 33342 and PI

1 × 10^6^ HCT116 cells were plated in 60 mm^2^ culture dishes and treated with increasing concentrations of DET (0.75, 1.5 and 3.0 µg/mL) while negative control was treated with the vehicle DMSO for 24 h. Then, the cells were collected, washed with PBS and proceed to staining with Hoechst 33342 (40 µg/mL) and propidium iodide solution (10 µg/mL) at room temperature in the dark for 30 min. The cells were then examined by inverted fluorescence microscopy (DM1600B, Leica, Wetzlar, Germany).

### 4.6. Externalization of Phosphatidylserine Detected by Annexin V and PI Staining

1 × 10^6^ HCT116 cells were plated in 60 mm^2^ culture dishes and exposed to different concentrations of DET (0.75, 1.5 and 3.0 µg/mL) for 24 h. Subsequently, cells were washed with PBS, resuspended in 1× Annexin V binding buffer and stained with Annexin V-fluorescein-isothiocyanate (FITC) (BD) and propidium iodide (PI) in the dark at room temperature for 15 min. 1× binding buffer was then added into each tube. The apoptotic and necrotic cell populations were then examined by flow cytometry [29]. The fluorescence intensity was detected in FL1-A (x-axis) and FL2-A channel (y-axis).

### 4.7. Induction of Cell Cycle Arrest

1 × 10^6^ HCT116 cells were plated in 60 mm^2^ culture dishes and exposed to different concentrations of DET (0.75, 1.5 and 3.0 µg/mL) for 24 h. The cells were harvested and centrifuged at 300× *g* at room temperature. The cells were then resuspended in 1 mL of buffer solution followed by centrifugation and washing with buffer solution. Subsequently, 250 µL of Solution A (trypsin buffer) was added and incubated for 10 min, followed by addition of 100 µL of Solution B (trypsin inhibitor and RNase buffer) and incubated at room temperature for 10 min. 200 µL of cold PI solution was added, gently mixed and incubated on ice for 10 min in the dark. Finally, samples were assessed by using flow cytometry (BD, San Jose, CA, USA).

### 4.8. Western Blot Analysis

1 × 10^6^ HCT116 cells were plated in 60 mm^2^ culture dishes and exposed to varying concentrations of DET (0.75, 1.5 and 3.0 µg/mL) for 24 h. The cells were collected, resuspended in cold RIPA buffer containing protease and phosphatase inhibitors and placed on ice for 5 min followed by centrifugation at 14,000× *g* for 15 min at 4 °C. 25 μg of total proteins was separated on the 12% SDS-PAGE gels and transferred onto nitrocellulose membranes, followed by the blocking using skim milk/bovine serum albumin for 1 h. Membranes were incubated with primary antibodies (cleaved PARP, cleaved caspase-3, p-p53, p21, CDK2, CDK4, CDK 6, cyclin D1, cyclin A2, cyclin B, cyclin E and GAPDH, Cell Signaling) at 4 °C overnight. Subsequently, the membranes were washed with TBST (0.05% Tween 20 in TBS) and incubated with corresponding anti-mouse/rabbit immunoglobulin G-horseradish peroxidase-conjugated secondary antibody (Cell Signaling, Danvers, MA, USA) at room temperature for 1 h. Then, the membranes were washed with TBST. The bands was detected by using enhanced chemiluminescence (ECL) detection kit (BioRad, Hercules, CA, USA) and visualized using gel documentation system. Protein bands were analyzed quantitatively and qualitatively with Vilber Lourmart.

### 4.9. Statistical Analysis

All the data were expressed as means ± standard error in every experiment. Statistical analysis was assessed by using ANOVA followed by Dunnett’s test or Student’s *t*-test in comparison with the respective control for each experiment, with *p* values < 0.05 being regarded as statistically significant.

## 5. Conclusions

In conclusion, our present findings have demonstrated growth inhibitory effect of DET inHCT116 cells. For the first time, our study highlighted the ability of DET as potential therapeutic agent to induce apoptosis and cell cycle arrest through regulation of p53, cyclins and cyclin dependent kinases proteins in HCT116 cells. Apoptosis was executed by DET via activation of caspase-3 and cleavage of PARP. Subsequently, execution of apoptosis was accompanied by a cell cycle arrest in S phase through activation of p53 and p21 in response to DNA damage. This is also indicated by the downregulation of CDK2, CDK4, cyclin B1, cyclin E2, cyclin D1 and cyclin A2 protein expression in HCT116 cells by DET. Therefore, our study suggests that DET is worthy of further investigations as a potential colorectal cancer therapy.

## Figures and Tables

**Table 1:** Comparison of the ^1^H- and ^13^C-NMR of isoDET and **1** (δ in ppm; 400 MHz in CDCl_3_).

Position	δ_H_, *J* (Hz) isoDET (But *et al.*, [24])	δ_H_, *J* (Hz) Compound 1	δ_C_ isoDET (But *et al.*, [24])	δ_C_ Compound 1
1	7.16, s	7.14, s	150.0	149.4
2	5.38, d (4.5)	5.37, d (4.5)	79.4	79.6
3a	2.39, dd (4.5, 14.0)	2.39, dd (4.5, 14.6)	40.0	40.2
3b	2.94, d (14.0)	2.93, d (14.6)		
4			135.4	135.5
5	5.13, d (10.0)	5.13, d (10.1)	125.2	125.5
6	5.17, d (10.0)	5.17, d (10.1)	78.7	78.8
7	3.15, m	3.15, m	49.8	50.0
8	4.53, ddd (4.0, 4.0, 12.0)	4.52, ddd (4.0, 4.0, 12.4)	74.0	74.1
9a	2.74, dd (4.0, 12.0)	2.74, dd (4.0, 12.4)	30.0	30.2
9b	3.06, dd (12.0, 12.0)	3.04, dd (12.0, 12.4)		
10			131.4	131.6
11			134.0	134.1
12			169.4	169.6
13a	5.65, d (3.2)	5.64, d (3.6)	123.0	123.3
13b	6.20, d (4.0)	6.20, d (4.1)		
14	1.78, s	1.78, s	21.5	21.7
15			174.3	174.4
16			166.5	166.7
17			135.4	135.5
18	1.93, s	1.93, s	18.1	18.3
19a	5.67, s	5.67, s	126.8	127.0
19b	6.15, s	6.14, s		

**Table 2:** Comparison of the ^1^H- and ^13^C-NMR of DET and **2** (δ in ppm; 400 MHz in CDCl_3_).

Position	δ_H_, *J* (Hz) DET (Than *et al.*, [25])	δ_H_, *J* (Hz) Compound 2	δ_C_ DET (Than *et al.*, [25])	δ_C_ Compound 2
1	7.08, br *s*	7.06, br *s*	153.5	153.3
2	5.46, td (1.8, 3.9)	5.46, td (1.8, 4.1)	81.4	81.4
3a	2.69, ddd (1.2, 2.1, 13.4)	2.70, ddd (1.2, 2.1, 13.4)	41.2	41.5
3b	2.85, dd (4.5, 13.8)	2.86, dd (4.6, 13.8)		
4			135.5	135.7
5	4.77, br d (10.5)	4.78, br d (10.5)	133.6	133.9
6	5.13, dd (8.1, 10.5)	5.14, dd (8.2, 10.5)	78.0	78.0
7	2.94, dt (3.6, 7.5)	2.94, dt (3.6, 7.7)	52.2	52.5
8	4.65, ddd (2.1, 3.6, 11.4)	4.65, ddd (1.8, 3.6, 11.6)	71.5	71.6
9a	2.78, d (12.3)	2.79, d (12.4)	33.4	33.7
9b	3.02, ddd (1.7, 3.0, 12.6)	3.01, ddd (1.7, 3.0, 12.5)		
10			128.3	128.7
11			134.0	134.1
12			169.3	169.4
13a	5.65, br d (3.3)	5.65, br d (3.2)	123.6	123.8
13b	6.23, br d (3.9)	6.23, br d (3.6)		
14	1.85, d (1.5)	1.84, d (1.4)	20.0	20.2
15			172.5	172.5
16			166.4	166.5
17			135.9	136.1
18	1.93, dd (1.2, 1.4)	1.93, s	18.2	18.3
19a	5.66, d (1.5)	5.66, d (1.8)	126.6	126.8
19b	6.14, t (1.2)	6.14, t (1.2)		

**Table 3:** IC_50_ values of deoxyelephantopin, isodeoxyelephantopin and 5-fluorouracil against HCT116 cancer cell line and CCD841-CoN normal cell line at 72 h.

Cell Lines	IC_50_ (µg/mL)		
	Deoxyelephantopin	Isodeoxyelephantopin	5-Fluorouracil ^a^
HCT116	0.73 ± 0.01	0.88 ± 0.02	0.73 ± 0.02
CCD841-CoN	21.69 ± 0.92	NA	>25

The data represented as mean ± S.E. of three independent experiments (*n* = 9). ND: Not determined. ^a^ 5-fluorouracil served as positive control.

## Abbreviations

The following abbreviations are used in this manuscript:

DET: deoxyelephantopin
IsoDET: isodeoxyelephantopin
